# Associations between generative AI–based pronunciation feedback and willingness to communicate in English: the mediating role of English pronunciation self-efficacy

**DOI:** 10.3389/fpsyg.2026.1918980

**Published:** 2026-08-13

**Authors:** Yang Lu, Yanchao Yang, Tianxue Cui, Zeyang Yang, Yuanyuan Cai, Bosheng Jing

**Affiliations:** 1 Faculty of International Languages, Qinggong College, North China University of Science and Technology, Tangshan, Hebei, China; 2 Institute of International Language Services Studies, Macau Millennium College, Macao, Macao SAR, China; 3 Center for Education and Exchange, Qinggong College, North China University of Science and Technology, Tangshan, Hebei, China; 4 College of Public Administration and Humanities, Dalian Maritime University, Dalian, Liaoning, China; 5 Department of Psychology, School of Education, Soochow University, Suzhou, China; 6 Department of Human Resources, Qinggong College, North China University of Science and Technology, Tangshan, China; 7 School of Humanities and Languages, The University of New South Wales, Sydney, NSW, Australia

**Keywords:** Chinese EFL students, English pronunciation self-efficacy, generative AI-based pronunciation feedback, mediation analysis, willingness to communicate

## Abstract

This study draws on Social Cognitive Theory to examine the association between learners’ perceptions of generative AI (GenAI)-based pronunciation feedback and their willingness to communicate (WTC) in English, with English pronunciation self-efficacy considered as a potential mediator. Using a cross-sectional survey design, data were collected from a convenience sample of 1,701 Chinese university EFL learners. Covariance-based structural equation modeling and bias-corrected bootstrapping were employed to test the hypothesized relationships and mediating effect. The results showed that positive perceptions of GenAI-based pronunciation feedback were significantly associated with greater WTC in English. English pronunciation self-efficacy partially mediated this association, with the indirect effect accounting for 69.9% of the total effect, while the direct effect remained significant. These findings suggest that pronunciation self-efficacy is an important, but not exclusive, psychological mechanism connecting perceptions of GenAI-based feedback with WTC. GenAI-based feedback may also relate to WTC through other mechanisms, such as reduced communication anxiety and enhanced psychological safety. By conceptualizing GenAI-based feedback as a technology-mediated environmental influence and pronunciation self-efficacy as a personal cognitive factor, this study extends the application of Social Cognitive Theory to the Chinese EFL context and offers new insight into how intelligent feedback technologies may support learners’ readiness to communicate in English.

## 1 Introduction

Willingness to communicate refers to an individual’s readiness to initiate communication in a specific situation rather than remain silent (McCroskey and Baer, 1985). It serves as an important psychological link between communicative competence and real-life communicative behavior, often influencing whether any interaction takes place. In the context of second language acquisition, this concept has been applied to L2 learning and is defined as L2 willingness to communicate, which refers to the readiness to engage in conversation at a given moment with a particular individual or group, using a second language (Macintyre et al., 1998). This definition highlights its context-dependent and dynamic character, combining relatively stable trait-like features with state-like fluctuations shaped by situational, interpersonal, and emotional factors. In English language learning contexts, L2 WTC, specifically the willingness to communicate in English, functions as a crucial psychological mechanism linking learners’ linguistic knowledge to its practical use in real interactions. Research consistently shows that L2 WTC in English is strongly associated with engagement (Wang and Chen, 2026), achievement (Al-Murtadha, 2021; Peng and MacIntyre, 2025), more frequent oral output, and increased overall use of the L2 (Hashimoto, 2002; Wang et al., 2020; Zhang et al., 2023). As a result, promoting learners’ willingness to communicate in English has become a key focus in modern second language acquisition research and foreign language teaching, carrying important implications for instructional design that seeks to facilitate genuine L2 interaction.

In the Chinese EFL context, learners generally have limited authentic and continuous opportunities to use English outside the classroom, which renders the classroom the main, or frequently the only, setting for both language input and output (Wang et al., 2021). In these settings, feedback from teachers or peers serves as a crucial external influence on learners’ emotional experiences, self-assessed competence, and motivation to communicate (Peng, 2020; Zare et al., 2022). Research shows that feedback that is supportive, precise, and practical can greatly enhance learners’ confidence (Tavakoli and Zarrinabadi, 2018), reduce language anxiety (Zarei and Rezadoust, 2020), and thereby enhance their willingness to engage in English communication. Therefore, feedback serves an essential role in offsetting the lack of authentic communicative contexts and in stimulating learners’ motivation to engage in L2 interactions.

With the ongoing transformation of education by artificial intelligence, generative AI (GenAI) tools are increasingly integrated into foreign language teaching, playing significant roles in both instruction and feedback (Zhong et al., 2025). In contrast to traditional human feedback, GenAI presents several distinct advantages: it delivers immediate responses, can be employed repeatedly without fatigue, provides highly personalized input, and tends to be perceived as less intimidating or judgmental (Zhong et al., 2025). These features make GenAI particularly useful for delivering continuous language support, especially in aspects such as speaking practice and error correction. Nevertheless, the majority of existing research has focused primarily on its role in enhancing linguistic accuracy (Yuvasri et al., 2025) or fluency (He et al., 2025). Far less attention has been given to its potential impact on learners’ willingness to communicate in English. Given that willingness to communicate is a proximal antecedent of actual second-language use, examining its association with learners’ perceptions of GenAI-based pronunciation feedback is essential for understanding the broader communicative value of such feedback.

Willingness to communicate in a foreign language has long been regarded as significantly influenced by self-efficacy (Kirkpatrick et al., 2025). In the context of English learning in China, pronunciation is not only widely perceived as a salient indicator of linguistic competence but also frequently serves as a key criterion for learners’ self-assessment (Yang, 2023). Owing to the effects of exam-focused education and limited opportunities for oral practice, many learners, despite having sufficient linguistic knowledge, hesitate to engage in authentic communication, fearing that their inaccurate pronunciation might impede intelligibility. This gives rise to the phenomenon of “mute English,” in which learners demonstrate strong writing skills but weak speaking abilities (Jin, 2025). Against this backdrop, English pronunciation self-efficacy (Yang, 2023) plays a crucial role in determining learners’ willingness to initiate spoken interaction.

Although previous research has found significant links between willingness to communicate and feedback (Jelínková et al., 2023; Zare et al., 2022), and between WTC and self-efficacy (Wang et al., 2025; Zare et al., 2022; Zhang and Dai, 2024), two key gaps remain. First, although general language self-efficacy has been extensively studied, pronunciation self-efficacy has been much less examined, particularly in the Chinese EFL context, where pronunciation is highly valued yet frequently a significant source of learner anxiety (Yang, 2023). Second, although feedback has been widely studied, empirical research specifically investigating feedback produced by generative artificial intelligence remains scarce. This gap is notable because GenAI offers a novel form of personalized, speech-based feedback that differs from traditional teacher or peer feedback in both its delivery method and the ways learners may perceive its authority and reliability.

Accordingly, this study seeks to examine the mediating role of English pronunciation self-efficacy in the relationship between generative AI–based pronunciation feedback and Chinese EFL learners’ willingness to communicate in English. Grounded in Social Cognitive Theory (Bandura, 1986), this study proposes that environmental factors, such as feedback from GenAI tools, can influence learners’ self-efficacy beliefs, which in turn affect their behavioral intentions, including their willingness to communicate in English.

## 2 Theoretical foundation and hypothesis development

Social Cognitive Theory proposes that human functioning results from reciprocal interactions among environmental influences, personal factors, and behavior (Bandura, 1986). In the present study, generative AI-based pronunciation feedback can be conceptualized as a technology-mediated environmental influence, English pronunciation self-efficacy as a domain-specific personal belief, and willingness to communicate as learners’ behavioral readiness to initiate English communication. According to Social Cognitive Theory, self-efficacy develops primarily through mastery experiences, vicarious experiences, verbal persuasion, and interpretations of physiological and affective states (Bandura, 1977, 1997). Generative AI-based pronunciation feedback may activate these sources by allowing learners to practice repeatedly, observe evidence of pronunciation improvement, compare their production with model pronunciations, and receive immediate and individualized corrective guidance. Its relatively private and low-pressure practice environment may also reduce pronunciation anxiety and fear of negative evaluation (Zhong et al., 2025). Consequently, learners who perceive generative AI feedback as useful, accurate, and supportive are likely to report stronger confidence in their ability to pronounce English intelligibly. Based on the above theoretical reasoning, generative AI-based pronunciation feedback is expected to be positively associated with learners’ English pronunciation self-efficacy. Therefore, the following hypothesis is proposed:

*H1*: Generative AI-based pronunciation feedback is positively associated with English pronunciation self-efficacy.

Social Cognitive Theory further maintains that self-efficacy beliefs shape individuals’ choices, effort, persistence, emotional reactions, and readiness to perform a particular behavior (Bandura, 1993). Learners with stronger English pronunciation self-efficacy may therefore be more willing to initiate and sustain English interactions because they anticipate greater communicative success and perceive less risk of misunderstanding or embarrassment. This explanation is consistent with the L2 WTC model, which identifies situation-specific communicative confidence as a proximal antecedent of willingness to communicate (Macintyre et al., 1998). Beyond this theoretical rationale, empirical studies have also demonstrated a close and positive relationship between self-efficacy and willingness to communicate (Wang et al., 2025; Zare et al., 2022; Zhang and Dai, 2024); therefore, the following hypothesis is proposed:

*H2*: English pronunciation self-efficacy is positively associated with willingness to communicate in English.

Meanwhile, generative AI-based pronunciation feedback may be directly associated with learners’ willingness to communicate. Immediate and individualized feedback can provide repeated opportunities for oral practice, reduce uncertainty about pronunciation accuracy, and create a relatively private and low-pressure environment for language use. Learners who perceive such feedback as useful, accurate, and supportive may therefore feel more prepared and willing to initiate English communication. Accordingly, the following hypothesis is proposed:

*H3*: Generative AI-based pronunciation feedback is positively associated with willingness to communicate in English.

**Figure 1:**
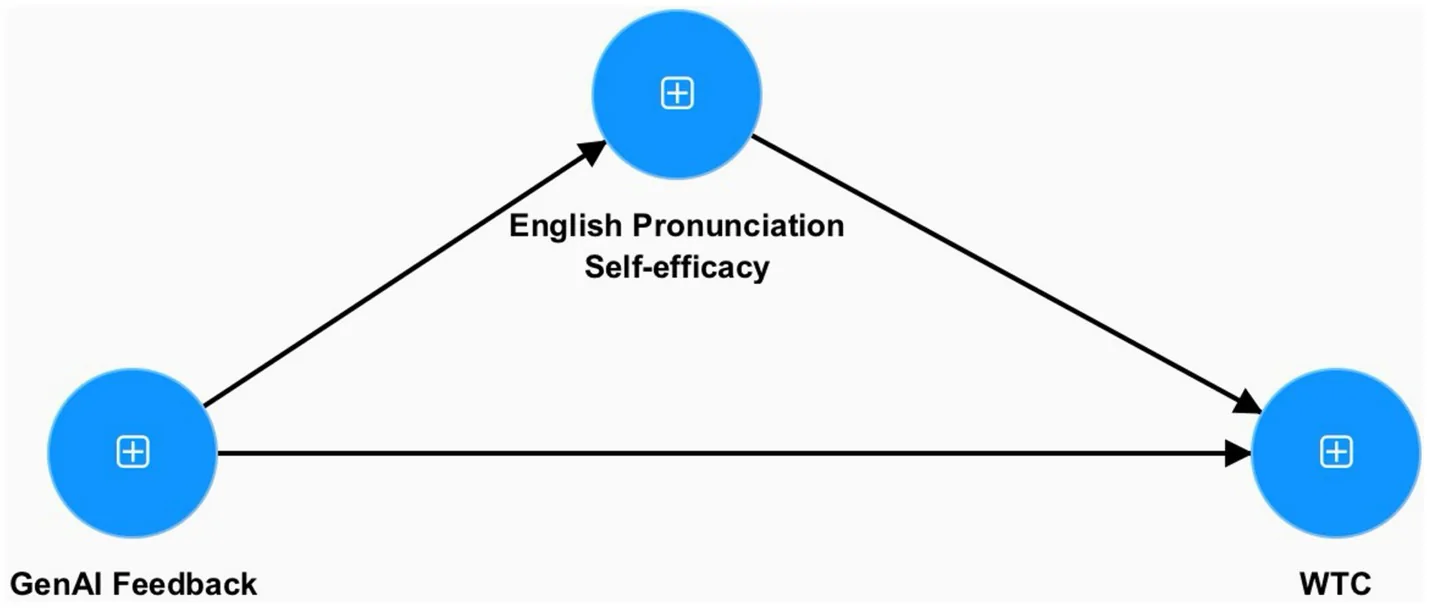
Conceptual model.

Based on the proposed hypotheses, the conceptual model of this study is presented in Figure 1.

## 3 Methods

### 3.1 Research design

This study adopted a quantitative, cross-sectional research design to examine the associations among learners’ perceptions of generative AI-based pronunciation feedback, English pronunciation self-efficacy, and willingness to communicate in English. Data were collected at a single point in time through a self-administered questionnaire administered to Chinese university students learning English as a foreign language. Structural equation modeling was employed to examine the hypothesized direct relationships among the three constructs and the indirect relationship through English pronunciation self-efficacy. As no variables were experimentally manipulated and participants were not randomly assigned to experimental conditions, the findings represent statistical associations rather than causal effects.

### 3.2 Participants

Convenience sampling was employed because a complete sampling frame of Chinese university EFL learners with experience of generative AI-based pronunciation feedback was not available. This sampling approach enabled the researchers to recruit eligible and willing participants efficiently through accessible university networks within the available time and resources. A total of 1,930 students accessed the online questionnaire. Of these, 175 chose not to participate, resulting in an initial sample of 1,755 responses and an effective response rate of 90.93%. After data screening, 54 responses were deemed invalid due to implausible age reports (i.e., participants reporting an age below 10, which falls outside the target population of tertiary education). Excluding these outliers left a final analytical sample of 1,701 valid responses. In terms of sex distribution, there were 794 males (46.7%) and 907 females (53.3%). Regarding participants’ birthplace, 422 (24.8%) were from urban areas, while the majority, 1,279 participants (75.2%), were from rural areas.

### 3.3 Instruments

#### 3.3.1 Scale for students’ attitudes toward AIGC feedback in English pronunciation learning

AIGC feedback in English pronunciation learning were measured using the Scale for Students’ Attitudes Toward AIGC Feedback in English Pronunciation Learning (Zhong et al., 2025). The scale comprises four dimensions: Accuracy, Strictness, Clarity, and Personalization, with each dimension consisting of four items, yielding a total of 16 items. Accuracy reflects students’ perceptions of how correctly the AIGC system identifies and diagnoses pronunciation errors. Strictness refers to the perceived rigor or stringency of the feedback provided by the AIGC tool. Clarity captures how clearly and understandably the feedback is presented. Personalization measures the extent to which students feel the feedback is tailored to their individual learning needs and progress. All items were rated on a five-point Likert scale ranging from 1 (strongly disagree) to 5 (strongly agree). To maintain model parsimony while preserving its multidimensional structure, the mean scores of Accuracy, Strictness, Clarity, and Personalization were used as theory-based composite indicators of the higher-order construct. This approach reduced the number of parameters in the structural model and was consistent with the study’s focus on construct-level relationships rather than item-level scale validation. Before calculating the dimension-level mean scores, the item-level measurement model was examined. The standardized factor loadings ranged from [0.942 to 0.963] for Accuracy, [0.942 to 0.951] for Strictness, [0.941 to 0.958] for Clarity, and [0.951 to 0.959] for Personalization, supporting the adequacy of the four dimensions. A sample item is: “The generative AI software is able to accurately identify individual phoneme errors in my pronunciation (such as vowel or consonant pronunciation mistakes).” The subscales demonstrated excellent internal consistency. For Accuracy, both Cronbach’s alpha and McDonald’s omega were 0.976. The corresponding coefficients were 0.972 for Strictness, 0.974 for Clarity, and 0.976 for Personalization.

#### 3.3.2 English pronunciation self-efficacy scale

The English Pronunciation Self-Efficacy Scale (Yang, 2023) is designed to measure students’ self-efficacy in English pronunciation and comprises two dimensions: Segmental Features (4 items) and Suprasegmental Features (5 items). The scale has been validated using both Rasch modeling and Classical Test Theory (CTT), demonstrating excellent reliability and validity. Moreover, it exhibits strong generalizability across demographic and contextual variables. All items were rated on a five-point Likert scale, with higher scores indicating greater self-efficacy in English pronunciation. A sample item is: “I think I can produce the sound of words according to their spelling and letter combination patterns.” The scale also demonstrated excellent internal consistency across its two dimensions. For Segmental Feature Pronunciation Self-Efficacy, both Cronbach’s alpha and McDonald’s omega were 0.967, whereas the corresponding coefficients for Suprasegmental Feature Pronunciation Self-Efficacy were 0.978.

#### 3.3.3 Willingness to communicate

Students’ willingness to communicate in English was measured using the L2 Willingness to Communicate (L2 WTC) scale (Lee et al., 2021). The scale uses the common question stem, “How much are you willing to communicate in English in this situation?,” followed by five specific communication situations, including speaking freely in an English class, speaking in front of the class, participating in a group discussion, giving a presentation to a large group, and explaining one’s own culture to classmates in English. For example, the first item, when combined with the common stem, asks: “How much are you willing to communicate in English when you are given a chance to talk freely in an English class?” Each item was rated on a five-point scale ranging from 1 (definitely not willing) to 5 (definitely willing). The scale demonstrated excellent internal consistency. Both Cronbach’s alpha and McDonald’s omega were 0.977, with identical 95% confidence intervals ranging from 0.975 to 0.978.

### 3.4 Analytical procedure

The data analysis followed a systematic, multi-step approach. First, preliminary analyses were conducted to examine the distributional characteristics of the data. Descriptive statistics, including means and standard deviations, were calculated for all variables. This was followed by Pearson product–moment correlation analyses to evaluate the bivariate relationships among GenAI–based pronunciation feedback, English pronunciation self-efficacy, and willingness to communicate in English.

Second, model fit was assessed using multiple indices, including the chi-square to degrees of freedom ratio (χ^2^/df), the Comparative Fit Index (CFI), the Tucker–Lewis Index (TLI), the Root Mean Square Error of Approximation (RMSEA), and the Standardized Root Mean Square Residual (SRMR). Following conventional recommendations, acceptable model fit was indicated by GFI, CFI and TLI values of 0.90 (Bentler and Bonett, 1980; Hu and Bentler, 1999), RMSEA value of 0.08 (Browne and Cudeck, 1992; Jöreskog and Sörbom, 1993), and SRMR value of 0.08 (Hu and Bentler, 1999). Although the chi-square statistic and its ratio to degrees of freedom were reported, they were not treated as the primary criteria for assessing model fit, due to the well-known sensitivity of the chi-square statistic to sample size (Babyak and Green, 2010). The measurement properties of the constructs were assessed sequentially using composite reliability (CR), average variance extracted (AVE), and the heterotrait–monotrait ratio of correlations (HTMT). CR was first examined to evaluate internal consistency reliability, with values of 0.70 or above considered acceptable. Convergent validity was then assessed using AVE, with values of 0.50 or higher indicating that a construct explained at least half of the variance in its indicators (Fornell and Larcker, 1981). Discriminant validity was evaluated using HTMT, with values below the conservative threshold of 0.85 indicating that the constructs were empirically distinct (Henseler et al., 2015).

Third, path analysis was performed to estimate the direct effects among the study variables. Parameter estimates were obtained using bias-corrected bootstrapping with 5,000 resamples. Direct effects were deemed statistically significant if the 95% bias-corrected bootstrap confidence intervals did not include zero (Hayes, 2022). Finally, mediation analysis was conducted to examine the indirect effect of GenAI–based pronunciation feedback on willingness to communicate in English through English pronunciation self-efficacy. The significance of the mediation was assessed using bias-corrected bootstrapping with 5,000 resamples, and mediation was considered present when the 95% confidence interval for the indirect effect did not include zero (Hayes, 2022).

### 3.5 Ethical considerations

This study was reviewed and approved by the Institutional Review Board of the first author’s affiliated institution (IRB No. QGXYLL20240003). All procedures adhered to the principles outlined in the Declaration of Helsinki. Convenience sampling was employed: teachers initially shared the questionnaire link with colleagues via WeChat and QQ work groups, who subsequently forwarded it to students. The survey was administered on the Wenjuanxing platform between December 3 and December 27, 2025, and required approximately 3–5 min to complete. Informed consent was obtained by providing a detailed explanation of the study’s purpose, the voluntary nature of participation, and strict data protection measures. No names or contact information were collected; only student IDs were recorded to enable matching with academic performance, and it was clearly stated that participation would not affect final course grades. All data were kept strictly confidential and used solely for academic research purposes. The first question asked participants whether they agreed to take part in the study; selecting “yes” indicated that they had read and understood the consent information and voluntarily consented to participate.

## 4 Results

### 4.1 Preliminary analyses

Descriptive statistics in Table 1 indicated that participants reported positive perceptions of AIGC-based pronunciation feedback, overall GenAI feedback (*M* = 4.13, SD = 0.79), specifically, accuracy (*M* = 4.15, SD = 0.82), strictness (*M* = 4.11, SD = 0.83), clarity (*M* = 4.14, SD = 0.81), and personalization (*M* = 4.13, SD = 0.81). Meanwhile, participants reported moderate level of English Pronunciation Self-efficacy (*M* = 3.75, SD = 0.87), specifically, Segmental pronunciation self-efficacy (*M* = 3.75, SD = 0.89), suprasegmental pronunciation self-efficacy (*M* = 3.75, SD = 0.90). In addition WTC (*M* = 3.77, SD = 0.86) was at moderate levels. Correlation analyses showed significant positive associations among three constructs (*r* = 0.562–0.739, *p* < 0.01).

**Table 1:** Descriptive and Pearson product–moment correlation results.

Dimension	*M*	SD	1	2	3	4	5	6	7	8	9
1. Accuracy	4.15	0.82	--								
2. Strictness	4.11	0.83	0.907^**^	--							
3. Clarity	4.14	0.81	0.906^**^	0.915^**^	--						
4. Personalization	4.13	0.81	0.886^**^	0.904^**^	0.940^**^	--					
5. GenAI Feedback	4.13	0.79	0.958^**^	0.965^**^	0.974^**^	0.966^**^	--				
6. Segment SE	3.75	0.89	0.533^**^	0.541^**^	0.547^**^	0.552^**^	0.563^**^	--			
7. Suprasegmental SE	3.75	0.90	0.548^**^	0.551^**^	0.567^**^	0.577^**^	0.580^**^	0.901^**^	--		
8. EPSE	3.75	0.87	0.555^**^	0.560^**^	0.572^**^	0.580^**^	0.587^**^	0.969^**^	0.981^**^	--	
9. WTC	3.77	0.86	0.541^**^	0.535^**^	0.547^**^	0.548^**^	0.562^**^	0.699^**^	0.738^**^	0.739^**^	--

** Correlation is significant at the 0.01 level (two-tailed). M = Mean, SD=Std. Deviation, SE = Self-efficacy, EPSE = English Pronunciation Self-efficacy.

### 4.2 Structural model fit

The hypothesized model was estimated using the covariance-based structural equation modeling (CB-SEM) module implemented in SmartPLS 4 (Ringle et al., 2024). The model yielded the following fit indices: χ^2^(41) = 385.639, *p* < 0.001; χ^2^/df = 9.406; RMSEA = 0.070 (90% CI [0.064, 0.077]); SRMR = 0.009; CFI = 0.988; TLI = 0.984; GFI = 0.960. The χ^2^/df value exceeded the conventional threshold of 3 to 5, indicating some degree of discrepancy between the hypothesized model and the observed data. However, chi-square-based indices are highly sensitive to sample size, and even relatively minor discrepancies may produce inflated values in a large sample such as that used in the present study (*N* = 1,701). In addition, although the use of dimension-level mean scores as composite observed indicators improved model parsimony, this aggregation did not explicitly model item-level measurement errors or residual covariances and may have contributed to some degree of model-data discrepancy. More importantly, the RMSEA was below 0.08, the SRMR was substantially below 0.08, and the CFI, TLI, and GFI all exceeded 0.95. Therefore, although the elevated χ^2^/df value warrants cautious interpretation, the remaining fit indices collectively support an acceptable overall fit of the hypothesized model to the observed data.

Table 2 summarizes the convergent validity, and discriminant validity of the three constructs. CR values ranged from 0.949 to 0.977, indicating high internal consistency. AVE values ranged from 0.893 to 0.909. The HTMT ratios ranged from 0.576 to 0.766. As all HTMT values were below 0.85, adequate discriminant validity was established among the three constructs.

**Table 2:** Convergent validity, and discriminant validity.

Construct	Convergent validity		Discriminant validity (HTMT)		
	CR	AVE	1	2	3
1. GenAI feedback	0.976	0.909	—		
2. EPSE	0.949	0.902	0.609	—	
3. WTC	0.977	0.893	0.576	0.766	—

### 4.3 Path analysis

**Figure 2:**
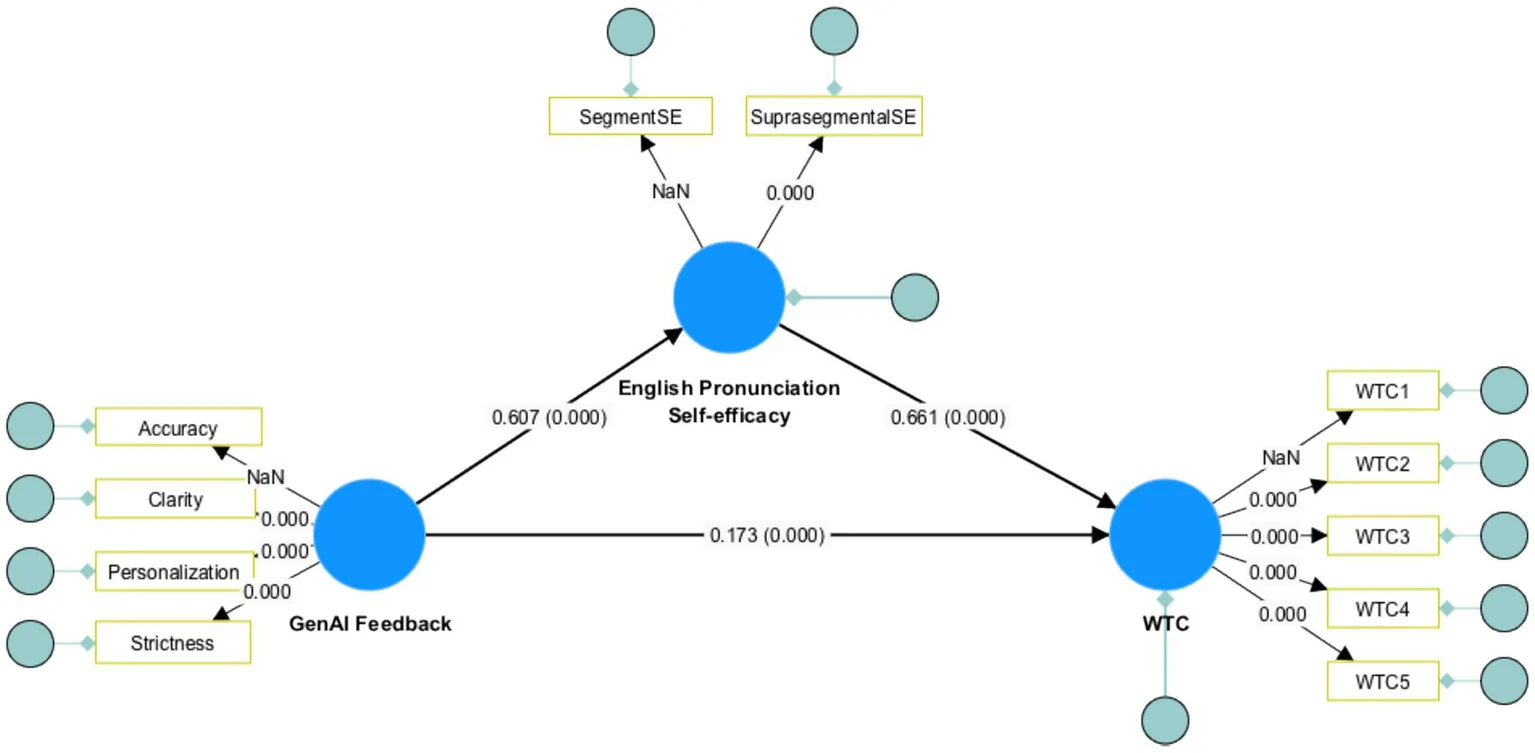
Structural equation model.

The path analysis among the study variables was conducted using bootstrapping procedures with bias-corrected 95% confidence intervals with 5,000 resamples. As shown in Table 3 and Figure 2, the path from EPSE to WTC was positive and statistically significant, *B* = 0.665, *β* = 0.661, 95% CI [0.610, 0.724], *p* < 0.001, indicating a strong association between EPSE and WTC. Similarly, GenAI feedback was positively associated with EPSE, *B* = 0.655, *β* = 0.607, 95% CI [0.606, 0.711], *p* < 0.001, indicating a strong association between GenAI and EPSE. A smaller but statistically significant positive association was also observed between GenAI feedback and WTC, *B* = 0. 188, *β* = 0. 173, 95% CI [0.137, 0.241], *p* < 0.001, indicating that these variables were linked even when accounting for the association via EPSE. Overall, all hypothesized direct paths were statistically significant, providing strong empirical support for the proposed structural relationships.

**Table 3:** Path analysis, mediation analysis and total effect analysis results.

Path	*B*	*β*	*p*	Bias corrected 95% CI		Type
				2.50%	97.50%	
EPSE - > WTC	0.665	0.661	<0.001	0.610	0.724	Direct effect
GenAI Feedback - > EPSE	0.655	0.607	<0.001	0.606	0.711	Direct effect
GenAI Feedback - > WTC	0.188	0.173	<0.001	0.137	0.241	Direct effect
GenAI Feedback - > EPSE - > WTC	0.436	0.401	<0.001	0.390	0.490	Indirect effect
GenAI Feedback - > WTC	0.624	0.574	<0.001	0.572	0.676	Total effect

B = unstandardized coefficient, β = standardized coefficient, CI=Confidence Interval.

### 4.4 Mediating analysis

The mediation effect of EPSE between GenAI feedback to WTC was examined using bootstrapped path analysis with 5,000 resamples. The results in Table 3 demonstrated that the mediation effect of EPSE was statistically significant, with *B* = 0.436 and *β* = 0.401. The bias-corrected 95% confidence interval did not include zero [0.390, 0.490], *p* < 0.001. This indicates that EPSE is meaningfully involved in the association between GenAI feedback and WTC within the model. Specifically, higher reported engagement with GenAI feedback was associated with higher EPSE, which in turn was associated with greater WTC.

### 4.5 Total effect

The results in Table 2 indicate that the total effect of GenAI Feedback on WTC is statistically significant (*B* = 0.624, *β* = 0.574, p < 0.001). The bias-corrected 95% confidence interval ranges from 0.572 to 0.676 and does not include zero, indicating a robust positive effect of GenAI Feedback on WTC. Furthermore, the mediation analysis shows that the indirect effect is 0.401, accounting for about 69.9% of the total effect, suggesting that the EPSE plays a substantial role in the relationship between GenAI Feedback and WTC.

## 5 Discussion

Drawing on Social Cognitive Theory, this study proposed a mediation model to investigate the role of English pronunciation self-efficacy in the relationship between GenAI feedback and willingness to communicate. This focus is particularly relevant in the Chinese EFL context, where “mute English” remains widespread: despite extensive formal instruction, many learners hesitate to speak not because of insufficient linguistic knowledge, but due to perceived low ability to produce English sounds accurately. The results showed that EPSE served as a significant partial mediator, explaining approximately 69.9% of the total standardized effect. This indicates that the impact of GenAI feedback on WTC is largely transmitted through learners’ enhanced self-efficacy in English pronunciation.

The findings resonate with a key principle of Social Cognitive Theory (Bandura, 1977, 1986), which posits that environmental factors, personal beliefs, and behavioral tendencies are meaningfully interconnected. In this study, GenAI feedback, an external, technology-mediated source of input, was associated with increased willingness to communicate largely through its effect on English pronunciation self-efficacy. This finding suggests that learners’ beliefs about their capacity to produce intelligible spoken English act as a key psychological mechanism linking AI-generated feedback to communicative intentions.

The current study identified a significant positive relationship between GenAI feedback and English pronunciation self-efficacy (EPSE), consistent with previous research demonstrating positive links between feedback-related aspects of the learning environment and learners’ self-efficacy in language learning contexts (Lu et al., 2022; Sherafati and Mahmoudi Largani, 2023; Yang et al., 2025). This relationship can be attributed to several distinctive characteristics of GenAI feedback, including its real-time delivery, targeted focus, and private mode of interaction (Zhong et al., 2025). Specifically, by rapidly analyzing learners’ speech, detecting pronunciation errors, and offering personalized acoustic models or corrective prompts, GenAI feedback can be seen as providing conditions that resemble mastery experiences (Bandura, 1997). By providing repeated opportunities to monitor their performance and witness incremental improvement, learners may develop a stronger sense of control over their pronunciation, which is closely linked to the formation of self-efficacy beliefs. This is consistent with previous research (Zhang et al., 2025), in which the responsiveness and personalization of GenAI positively predicted EFL learners’ AI trust and AI learning self-efficacy, which, in turn, were positively associated with their learning engagement. Although AI learning self-efficacy differs from the pronunciation-specific self-efficacy examined in the present study, their findings provide parallel empirical evidence that the educational value of GenAI features may operate through learners’ psychological beliefs. In the context of pronunciation learning, responsive and personalized GenAI feedback may similarly strengthen learners’ confidence in their pronunciation capabilities, thereby increasing their willingness to communicate in English. Additionally, the private and non-judgmental nature of GenAI-mediated practice can help reduce emotional arousal, such as anxiety or fear of embarrassment, which is another important factor shaping self-efficacy judgments (Bandura, 1997). This dynamic is especially pertinent in the Chinese EFL context, where “mute English” frequently stems from pronunciation-related anxiety and worries about “losing face” during public speaking. The low-pressure, self-paced environment provided by GenAI tools enables learners to engage in repeated oral practice without social evaluation, thereby enhancing their sense of control over pronunciation skills.

The results further showed a significant positive relationship between GenAI feedback and willingness to communicate, even after considering the mediating effect of pronunciation self-efficacy. This finding is consistent with previous research indicating that supportive, responsive input, whether from teachers, peers, or digital tools, is generally associated with higher levels of communicative engagement in second language learning (Jelínková et al., 2023; Tavakoli and Zarrinabadi, 2018). One possible explanation is that GenAI feedback provides a psychologically safe and controllable interactional environment, enabling learners to experiment with the language without fear of negative evaluation. Consequently, learners may feel more willing to initiate communication when they perceive fewer affective or interpersonal barriers in the immediate context.

The results showed that English pronunciation self-efficacy was significantly and positively related to willingness to communicate in this study. This finding aligns with extensive prior research indicating that self-efficacy, especially in speaking-related domains, is generally associated with a higher readiness to participate in oral communication (Kirkpatrick et al., 2025; Liu Z. et al., 2025; Zhang and Dai, 2024). While most prior research has focused on general speaking or communicative self-efficacy as a broad construct, the present study specifically examines English pronunciation self-efficacy, given its particular relevance to the “mute English” phenomenon among Chinese EFL learners. Due to limited exposure to authentic spoken input and few opportunities for low-stakes practice, many learners perceive their English pronunciation as inadequate or unintelligible. This perception often leads to low confidence, which in turn contributes to avoidance of speaking situations to prevent embarrassment or loss of face. The strong relationship between EPSE and willingness to communicate observed in this study highlights a central psychological mechanism underlying “mute English”: learners who feel more competent in their pronunciation are more likely to initiate and maintain spoken interaction. More importantly, the mediation analysis showed that EPSE partially, rather than fully, mediated the association between learners’ perceptions of GenAI-based pronunciation feedback and WTC. The indirect effect through EPSE accounted for 69.9% of the total effect, while the direct association between GenAI-based feedback perceptions and WTC remained significant. This partial mediation suggests that EPSE is a primary, but not exclusive, mechanism connecting perceptions of GenAI-based pronunciation feedback with WTC. Other psychological variables, such as learners’ attitudes toward GenAI-assisted language learning, L2 resilience, learning motivation, and foreign language enjoyment, may also help explain the remaining direct association. For instance, drawing on previous research (Liu H. et al., 2025), GenAI feedback may also promote WTC by reducing anxiety and satisfying learners’ autonomy needs, which should be examined in future research.

## 6 Implications

The findings of this study have significant theoretical, practical, and technological implications for second language teaching, technology integration, and learner psychology. Traditionally, effective feedback in L2 learning has been linked primarily to human sources, such as teachers or peers, whose responsiveness and support are known to strengthen learners’ perceived confidence and willingness to communicate. The present findings extend this understanding by demonstrating that feedback from generative AI can likewise enhance learners’ pronunciation self-efficacy and foster greater willingness to communicate in English.

From a practical perspective, in the Chinese EFL context, where “mute English” continues to be a persistent challenge, the use of generative AI for pronunciation feedback represents a timely and promising pedagogical innovation. Unlike traditional classroom settings, where corrective feedback on pronunciation is often limited, delayed, or avoided to preserve learners’ face, AI-powered tools offer immediate, private, and non-judgmental practice environments. These features can help reduce learners’ sensitivity to negative evaluation and face-threatening concerns, thereby lowering affective barriers to oral participation. Consequently, educators may consider incorporating generative AI–based pronunciation tools to provide learners with additional low-stakes speaking opportunities outside the classroom, particularly for those who experience high levels of evaluation anxiety.

From a technological perspective, these findings emphasize the importance of designing educational GenAI systems that go beyond mere error detection to incorporate emotionally intelligent feedback. To effectively support learners’ willingness to communicate, pronunciation tools should not only identify phonological errors but also provide feedback that enhances learner confidence, using encouraging language, offering actionable guidance, and adapting to individual proficiency levels. Ultimately, AI developers should prioritize affective usability alongside technical accuracy, ensuring that these tools function not merely as evaluators, but as supportive conversational partners that empower learners to speak with confidence.

## 7 Limitations and suggestions

Several limitations of the present study should be noted. First, the sample was drawn from a single institution, which may limit the generalizability of the findings. Results obtained from a relatively homogeneous population may not reflect broader or more diverse educational contexts. To address this limitation, future research should employ stratified, multi-site sampling strategies to test the robustness and boundary conditions of the observed effects.

Second, although the study is theoretically grounded in Social Cognitive Theory, which frames human functioning as a dynamic system of reciprocal interactions among personal, behavioral, and environmental factors, the cross-sectional design precludes conclusions about directionality, feedback mechanisms, or developmental trajectories. A more rigorous empirical examination of Social Cognitive Theory would therefore require longitudinal panel designs with repeated measurements (e.g., cross-lagged panel models or latent growth modeling) or experimental interventions capable of isolating causal pathways and reciprocal dynamics.

Moreover, this study employed convenience sampling, which may have introduced selection bias because participants who were readily accessible and willing to participate may differ from the broader population of Chinese university EFL learners. Therefore, the sample may not fully represent students from different geographical regions, types of universities, educational backgrounds, or levels of English proficiency, which limits the generalizability of the findings. Future studies should employ probability-based sampling methods, such as stratified random sampling, and recruit participants from a wider range of universities and regions to examine the robustness of the present findings.

Additionally, the data were collected from students at a single institution using self-report questionnaires administered at the same time. Therefore, the possibility of common method bias cannot be completely ruled out. This bias may have inflated the observed associations among perceptions of generative AI-based pronunciation feedback, English pronunciation self-efficacy, and willingness to communicate in English. Accordingly, the results should be interpreted with caution. Future research should recruit participants from multiple institutions and employ procedural or statistical remedies, such as temporal separation, multiple data sources, or a marker-variable technique, to minimize and assess potential common method bias.

Furthermore, the cross-sectional design and single-time-point measurement of the present study prevent the establishment of temporal ordering and causal direction among perceptions of GenAI-based pronunciation feedback, EPSE, and WTC. Future research should adopt a multi-wave longitudinal design in which all focal variables are repeatedly measured at multiple time points. Such data would allow researchers to employ cross-lagged panel modeling to examine autoregressive and cross-lagged relationships and provide stronger evidence regarding the temporal and directional relationships among the variables.

In addition, several potentially important confounding variables were not controlled for in the present study. For example, language anxiety may be negatively associated with both EPSE and WTC, whereas higher prior pronunciation proficiency may contribute to stronger pronunciation self-efficacy and greater communicative readiness. Familiarity with GenAI technology may also be associated with more positive perceptions of GenAI-based feedback and greater willingness to use such tools for oral practice. Future studies should measure and statistically control for these variables to obtain more precise estimates of the relationships among GenAI feedback perceptions, EPSE, and WTC.

Finally, the study did not comprehensively account for all potential confounding variables. The lack of rigorous control over extraneous factors may have obscured the true relationships among the primary variables, potentially resulting in spurious findings. Future research should theoretically justify and empirically control for a broad range of confounders through careful study design, thereby producing more precise and less biased estimates of the associations under investigation.

## 8 Conclusion

This study investigated the relationships between generative AI–based pronunciation feedback and learners’ willingness to communicate in English, with particular attention to the mediating role of English pronunciation self-efficacy. The results indicate that learners’ positive perceptions of GenAI-based pronunciation feedback were associated with greater communicative readiness primarily through enhanced pronunciation self-efficacy, with the indirect effect accounting for 69.9% of the total effect, while the remaining direct effect, although significant, was comparatively modest (*β* = 0.173). Framing these relationships within a social cognitive perspective highlights the complex interplay among technological affordances, learner beliefs, and communicative behavior in EFL contexts. Despite the study’s methodological limitations, the findings underscore the potential of generative AI–supported pronunciation feedback as a valuable tool for promoting oral engagement, particularly in settings where low-stakes opportunities for spoken practice are limited.

## Data Availability

The raw data supporting the conclusions of this article will be made available by the authors, without undue reservation.
