# Supplementary material for: Associations between generative AI–based pronunciation feedback and willingness to communicate in English: the mediating role of English pronunciation self-efficacy
Source: Front Psychol. 2026 Aug 13;17:1918980. doi: 10.3389/fpsyg.2026.1918980 (PMC13518164; doi:10.3389/fpsyg.2026.1918980)
Supplement: Supplementary file 1 [file Data_Sheet_1.PDF]

L2 WTC

*How much are you willing to communicate in English in this situation?

1. Definitely not willing; 2. Probably not willing; 3. Perhaps willing; 4. Probably willing; and 5. Definitely willing

1. When you are given a chance to talk freely in an English class
2. When you have a chance to talk in front of the class in an English class
3. When you have a group discussion in an English class
4. When you have a chance to make a presentation in front of a large group
5. When you have a chance to explain your own culture in English to your classmates

English Pronunciation Self-efficacy

Segmental Features

1. I think I can pronounce the phoneme represented by each phonetic symbol correctly.

1. I think I can produce the sound of words according to their spelling and letter combination patterns.

2. I think I can determine the correct pronunciation of a polysyllabic word in context (e.g. present, noun: [ˈprez.ənt] verb: [prɪˈzent]).

3. I think I can pronounce a new word correctly according to its phonetic symbols.

4. I think I can read aloud a passage in a rhythmic manner with a clear sense of stress.

Suprasegmental Features

5. I think I can read English texts with the necessary liaison.

6. I think I can change the pronunciation of certain phonemes (e.g. [t] in stop) when reading English texts, depending on the influence of the preceding and following phonemes.

7. I think I can recognize clusters of meaning according to context and use pauses correctly to avoid ambiguity.

8. I think I can use appropriate intonation to express different attitudes, feelings and intentions.

Scale for Students' Attitude towards AIGC Feedback in English Pronunciation Learning

Accuracy

A1: The generative AI software is able to accurately identify individual phoneme errors in my pronunciation (such as vowel or consonant pronunciation mistakes).

A2: The generative AI software can distinguish my errors in sentence stress and word stress, providing specific corrections.

A3: The generative AI software can correctly identify intonation errors in my speech when expressing emotions, attitudes, or intentions in different contexts and provide targeted feedback.

A4: The generative AI software can correctly detect connected speech or pause errors in my pronunciation and offer relevant feedback and suggestions.

Strictness

S1: Any error, no matter how small, found in my pronunciation by the generative AI software will be strictly pointed out, with suggestions for correction.

S2:If I repeat the same pronunciation error, the generative AI software will correct it strictly each time until I completely correct it.

S3:Even the smallest pronunciation errors will be rigorously corrected by the generative AI software, without any tolerance.

S4:The generative AI software takes correction very seriously, repeatedly confirming whether I have mastered the pronunciation correctly.

Clarity

C1: The terminology and language used by the generative AI software are clear and easy to understand, enabling me to easily follow its suggestions for improvement.

C2: The generative AI software uses simple demonstrations and examples to help me better understand its pronunciation correction suggestions.

C3: The feedback steps provided by the generative AI software are clear and well-defined, with each step being easy for me to follow.

C4: The feedback from the generative AI software is very intuitive, allowing me to understand and follow its suggestions without needing further explanation or help.

Personalization

P1: The generative AI software remembers my previous pronunciation issues and offers more in-depth corrections on these issues in subsequent feedback.

P2: Based on my past learning records, the generative AI software provides pronunciation practice suggestions that better meet my personal needs.

P3: The feedback provided by the generative AI software seems tailor-made for me, based on my past performance and unique pronunciation issues.

P4: The generative AI software remembers my preferences (e.g., feedback style or practice type) and reflects these preferences in future interactions.
